# Time-lapse confocal imaging datasets to assess structural and dynamic properties of subcellular nanostructures

**DOI:** 10.1038/sdata.2018.191

**Published:** 2018-09-18

**Authors:** Gianmarco Ferri, Luca Digiacomo, Francesca D’Autilia, William Durso, Giulio Caracciolo, Francesco Cardarelli

**Affiliations:** 1 NEST-Scuola Normale Superiore, Istituto Nanoscienze-CNR (CNR-NANO), 56127 Pisa, Italy; 2 Nanoscopy, Nanophysics, Istituto Italiano di Tecnologia, via Morego 30, 16163 Genoa, Italy; 3 Department of Molecular Medicine, "La Sapienza" University of Rome, 00161 Rome, Italy; 4 Center for Nanotechnology Innovation@NEST, Istituto Italiano di Tecnologia, 56127 Pisa, Italy

**Keywords:** Imaging and sensing, Biological fluorescence, Organelles

## Abstract

Time-lapse optical microscopy datasets from living cells can potentially afford an enormous amount of quantitative information on the relevant structural and dynamic properties of sub-cellular organelles/structures, provided that both the spatial and temporal dimensions are properly sampled during the experiment. Here we provide exemplary live-cell, time-lapse confocal imaging datasets corresponding to three sub-cellular structures of the endo-lysosomal pathway, i.e. early endosomes, late endosomes and lysosomes, along with detailed guidelines to produce analogous experiments. Validation of the datasets is conducted by means of established analytical tools to extract the structural and dynamic properties at the sub-cellular scale, such as Single Particle Tracking (SPT) and imaging derived Mean Square Displacement (*i*MSD) analyses. In our aim, the present work would help other researchers in the field to reuse the provided datasets for their own scopes, and to combine their creative approaches/analyses to similar acquisitions.

## Background & Summary

Membrane-enclosed, sub-micrometric, and dynamic organelles or compartments, such as endocytic/secretory vesicles, early-late endosomes, lysosomes, mitochondria, etc. are emerging as a legitimate platform for cell-signaling regulation^1,2^. A growing body of evidences, in fact, support the idea that a finely tuned regulation of their *structural* (e.g. size/morphology) and *dynamic* (e.g. diffusivity, mode of motion) properties determines how cells comply with internal or external stimuli^3–5^. As a consequence, average alterations of the same properties are typically found as hallmarks of pathological conditions. A few examples include derailed endocytosis commonly found in cancer cells^3,4^, altered granule structural and trafficking properties characterize β-cells exposed to Type-2-Diabetes-mimicking conditions^6^, enlarged lysosomes packed with twisted microtubules found in globoid cell leukodystrophy or galactosylceramide lipidosis^7^and abnormalities in the endosomal-lysosomal system observed in neurodegenerative diseases, especially Alzheimer’s disease (AD)^8^.

Concerning structural information, current knowledge mostly owes to transmission electron microscopy (TEM) analysis. Unfortunately, however, the peculiar nanoscale spatial accuracy of TEM is achieved at the expenses of the dynamic information, which is inevitably lost. On the other hand, the recent advances in live-cell imaging technologies, including development of specific fluorescent markers, optimization of labeling protocols, and availability of ever more sensitive and less invasive optical microscopes potentially open the way to the study of structural and dynamic properties at the nano-micro scale and in live cells. Along with this, well established analytical tools are at our disposal to extract the relevant structural (e.g. size by phasor analysis of local image correlation spectroscopy, PLICS^9^) or dynamic (e.g. diffusion law by SPT^10–15^) parameters at the sub-cellular scale. In this context, some of us recently introduced an additional tool based on spatiotemporal fluctuation analysis, that is able to simultaneously extract both the structural and dynamic properties of diffusing objects directly from standard imaging, with no need for preliminarily assumptions/knowledge on the system and no need to extract the single trajectories (for applications on molecules/nanoparticles see refs 16–19, for applications on sub-cellular organelles/structures see ref. 20).

**Figure 1:**
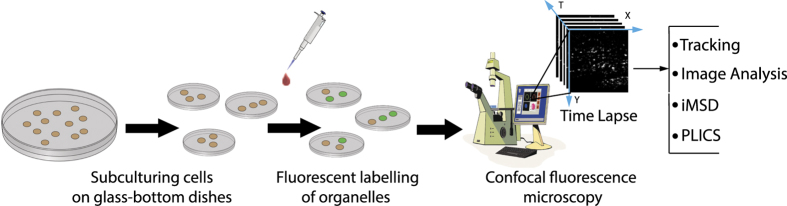
Schematic workflow of the method. Cells were plated 48-h before the experiment onto dishes suitable for confocal microscopy. 24 h before the experiment, cells were transiently transfected with specific proteins to label the cytoplasmic organelle of interest (e.g. early and late endosomes). In the case of lysosomes, 20 min before the experiment, cell were incubated with Lysotrackercontaining medium. A typical confocal microscopy experiment consists in the acquisition of a stack of images (time lapse) of a cytoplasmic portion of a labelled cell, with experimental parameters (temporal resolution, pixel size, etc.) set to the optimal values (see Technical Validation section) for further analysis (e.g. localization and tracking, iMSD, image analysis).

The overall emerging picture in the field of cell biophysics, as discussed in more detail elsewhere (see ref. 21), is that proper sampling of the spatial and temporal dimensions can enhance the performances of current optical microscopy methods and may provide further insight into biological processes of relevance. The general workflow from sample preparation to time-lapse imaging of intracellular nanostructures and analysis for the derivation of their structural/dynamic properties is presented in Fig. 1. Here we make three exemplary datasets (corresponding to three intracellular organelles, i.e. the lysosomes, the early and late endosomes) publicly available (Data Citation 1) alongside datasets validation and detailed guidelines to perform analogous experiments. In addition, we provide the data analysis algorithm to extract the *i*MSD from imaging. In our hopes, this would help other researchers to reuse the data for their own scopes, and to combine their creative approaches to the proposed platform, thus enabling quantitative screening of key sub-cellular processes. Notably, provided that the key experimental parameters (i.e. frame rate, pixel size, low bleaching, labelling procedure, etc. proper time window) are kept constant, the data provided here can be considered as an invariant reference for reuse or comparison with analogous datasets acquired, for instance, under different experimental conditions (e.g. in different cell lines, culturing conditions, pathological states, etc.). As such, the present contribution it is likely to become a valuable resource for researchers interested in the statistical characterization of the diversity of structural and dynamic properties of sub-cellular components and the effects of internal/external stimuli on these properties.

## Methods

These methods are expanded versions of descriptions in our related work (ref. 20).

### Sample preparation

A distinguishing feature of the approach presented here is that it is thought to work on living cells/samples. Thus, any experiment should start with the specific procedures devoted to maintaining the sample under ideal growing/culturing conditions. In the case presented here, HeLa cells (CCL-2 ATCC) were cultured in Dulbecco's modified eagle medium (DMEM) without phenol red (Gibco), supplemented with 10% fetal bovine serum (FBS, Gibco), 100 U/mL of penicillin, and 100 μg/mL of streptomycin in a humidified incubator at 37 °C and 5% CO_2_. Cells were seeded on 22-mm glass bottom dishes (WillCo Wells) and allowed to adhere overnight in a 37 °C and 5% CO_2_ cell culture incubator. CellLight Early Endosomes-GFP BacMam 2.0 (Life Technologies) was used to mark early endosomes, while CellLight Late Endosomes-GFP BacMan 2.0 (Life Technologies) was used to mark late endosomes Cells were transduced with CellLight reagents according to the manufacturer’s instructions. Briefly, cells were incubated with 40 μl of the CellLight solution with baculovirus in full growth medium overnight at 37 °C and 5% CO_2_, the day before the experiment. Staining with LysoTracker Red DND-99 (Life Technologies) was performed starting from a stock solution diluted to a final concentration of 60 nM in fresh growth medium. The medium was removed from the dish and pre-warmed (37 °C) LysoTracker-containing medium was added. Cells were incubated for 20 minutes with LysoTracker-containing medium, then washed and observed.

### Live-cell confocal imaging

Confocal fluorescence image series were acquired with an Olympus FluoView FV1000 confocal microscope with a 60x NA 1.20 water immersion objective. All experiments were carried out at 37 °C and 5% CO_2_ using an incubation chamber enclosing the microscope stage and body. 488 nm Argon laser was used for excitation of early and late endosomes. The fluorescence emission was collected between 500 and 600 nm with the PMT detector in analog mode. 543 nm HeNe laser was used to excite Lysotracker. In this case, fluorescence emission was collected between 555 and 655 nm with the PMT detector in analog mode. The diameter of the detection pinhole was set to the size of 1 Airy. A series of sequential images at 16 bits were collected at a fixed pixel size of 69 nm selecting a region of interest of 256x256 pixels within the cell (i.e. corresponding approximately to 17x17μm). The imaging conditions are reported in Table 1. In detail, for early and late endosomes, we used a pixel-dwell time of 2 μs that generates in turn a frame time of 129 ms (N=1000 frames were acquired). For lysosomes we set a pixel-dwell time of 1 μs that yields a frame time of 69 ms (N=400 frames were acquired).

### Code availability

Both the *i*MSD processing of the acquired image-stacks and the subsequent data analysis were carried out with custom scripts working in MATLAB (MathWorks Inc., Natick, MA). The code for *i*MSD calculation and fitting is made available (Data Citation 1). Analogous analytical tools are embedded into the SimFCS software that can be downloaded at: https://www.lfd.uci.edu/globals/.

### Trajectory analysis

**Figure 2:**
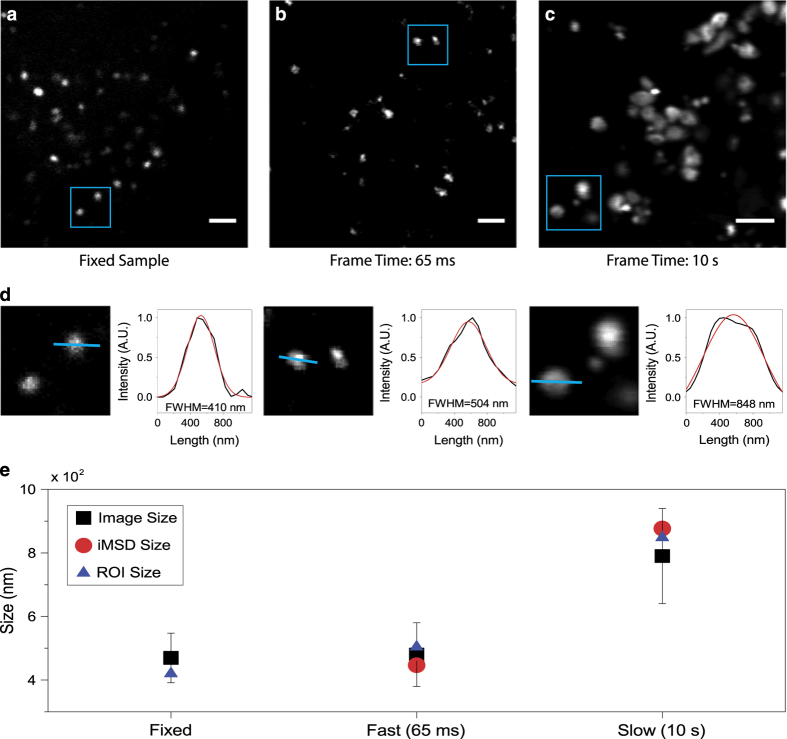
Setting the experimental parameters. (**a**) Exemplary image of stained lysosomes in a fixed sample. Scale bar: 2 μm (**b**) First frame of a stack of images of stained lysosomes in a living cell, acquired with the appropriate parameters. Temporal resolution: 65 ms/frame. Scale bar: 2 μm (**c**) First frame of a stack of images of stained lysosomes in a living cell, acquired at low speed: artifactual deformation of the apparent lysosome size due to organell motion during imaging is clearly visible. Temporal resolution: 10 s/frame. Scale bar: 2 μm. (**d**) Example of size calculation for imaged lysosomes in blue ROI of (**a**), (**b**) and (**c**). The intensity profile along the blue line was fitted with a Gaussian function to retrieve the FWHM, i.e. an estimate of spot size. FWHM values are reported for each fitting. (**e**) Graphical representation of size values obtained by image analysis described in panel (**d**) for all imaged lysosomes (black square, mean value and standard deviation), for lysosomes enclosed within the blue ROI (blue triangle) and retrieved by iMSD analysis (red circle).

Trajectory analysis (Fig. 2) was performed using TrackMate plugin in ImageJ, applied to the ee02 dataset available in Data Citation 1. Briefly, the LogDetector algorithm was used to detect the fluorescence spot; the Lap Tracker algorithm was used to perform the analysis of trajectories; finally, by using the filter based on the temporal duration, only trajectories longer than 12 s were retained. The MSD was calculated for each retained trajectory by using the ‘msdanalyzer’ matlab tool, available at: https://github.com/tinevez/msdanalyzer.

### Data records

The data records associated with this paper are available at the figshare repository (Data Citation 1). They are subdivided into three folders: ‘Early Endosomes’ (ee), ‘Late Endosomes’ (le) and ‘Lysosomes’ (ly), as summarized in Table 1. Each folder contains sub-folders relative to single live-cell acquisitions (performed as described in Methods): N=40 for early and late endosomes, 26 for lysosomes. Each acquisition is composed of a time-lapse stack of 1000 images (for early and late endosomes) and 460 for lysosomes in *.tif* format, 130 KB each. Images are named according to the name of the organelle and the progressive number of frames (e.g.: for the first ‘early endosome’ acquisition, “ee1_xxxx” where xxxx is the frame number, from 0001 to 1000). Data records contain also single excel files containing the typical output of one of the available data-analysis algorithms (i.e. *i*MSD), and are named according to the imaged organelle/structure (e.g. “ee1.xls”). For more details refer to the ‘Usage notes’ section. Finally, an excel file composed by three data sheets (one for each analyzed organelle/structure) contains a summary of all the structural and dynamic parameters extracted from *i*MSD analysis.

## Technical Validation

CellLight Early Endosomes-GFP, CellLight Late Endosomes-GFP, from Thermo Fisher/Invitrogen are well-established and characterized systems based on baculovirus endocytosis (BacMam 2.0). They are used here to provide specific labeling of intracellular early endosomes and late endosomes, respectively. For Early Endosomes labelling, the expression vector contains the fusion construct Rab5a-GFP, while for Late Endosomes labelling, the expression vector contains the Rab7-GFP fusion construct. LysoTracker Red DND-99 (Termo Fisher/Invitrogen) is an organic dye for live cell labelling of acidic organelles, commonly used for labelling lysosomes. The Olympus FV1000 microscope used here (and in previous work^20^) was maintained under technical and professional conditions. The point spread function (PSF) was calibrated using 30-nm fluorescent beads and resulted to be about 220 nm at 488-nm excitation and 250 nm at 561-nm excitation. The microscope is equipped with a temperature and CO_2_ control unit (OkoLab) that ensures proper conditions for live cell imaging.

Validation of time-lapse experiments is presented here through the calculation of the characteristic size of the diffusing object (the lysosome in the example reported in Fig. 2), under the following assumption: if the time resolution of imaging is appropriate to describe the motion of the organelle under study, this latter will appear as ‘immobile’ within each captured frame, i.e. it will display a characteristic size that, on average, is not deformed due to the imaging speed. A limit condition, in this regard, can be reached by fixing the sample, i.e. by artificially immobilizing the organelle of interest. This condition can be used as a reference to obtain the expected organelle size under the imaging conditions chosen (e.g. laser wavelength, pixel size, objective, etc.). This is shown in Fig. 2a-c for lysosomes, along with an acquisition performed in live cells at the desired temporal resolution (i.e. 65 ms/frame, Fig. 2b), and an acquisition performed intentionally at slow speed (i.e. 10 sec/frame, Fig. 2c). Thus, we show quantification of organelles sizes directly from the optical microscopy images (Fig. 2d). In particular, the size of each organelle/structure in the image is extracted by means of ImageJ software. For each spot of imaged lysosomes, intensity profile is plotted and fitted with a Gaussian function to obtain full width at half maximum (FWHM) value that can be assumed as estimation of spot diameter. As expected (Fig. 2e), the acquisition performed at an appropriate temporal resolution (65 ms, in this case) yields a characteristic size of the structure of interest that closely resembles that obtained from the fixed sample, either by using the standard tool described above or the *i*MSD y-axis intercept. By contrast, the artificially slow acquisition yields a substantially enlarged apparent size of the same structure, due to its substantial movement during imaging. It is clear that, under these latter experimental conditions, both the structural and dynamic information embedded into the time-lapse acquisition do not faithfully describe the intrinsic properties of the structure of interest. Concerning the spatial dimension, it is suggested to oversample the pixel by the available PSF of the laser beam, i.e. set a pixel size at least 3–5 times smaller than the PSF.

## Usage Notes

**Figure 3:**
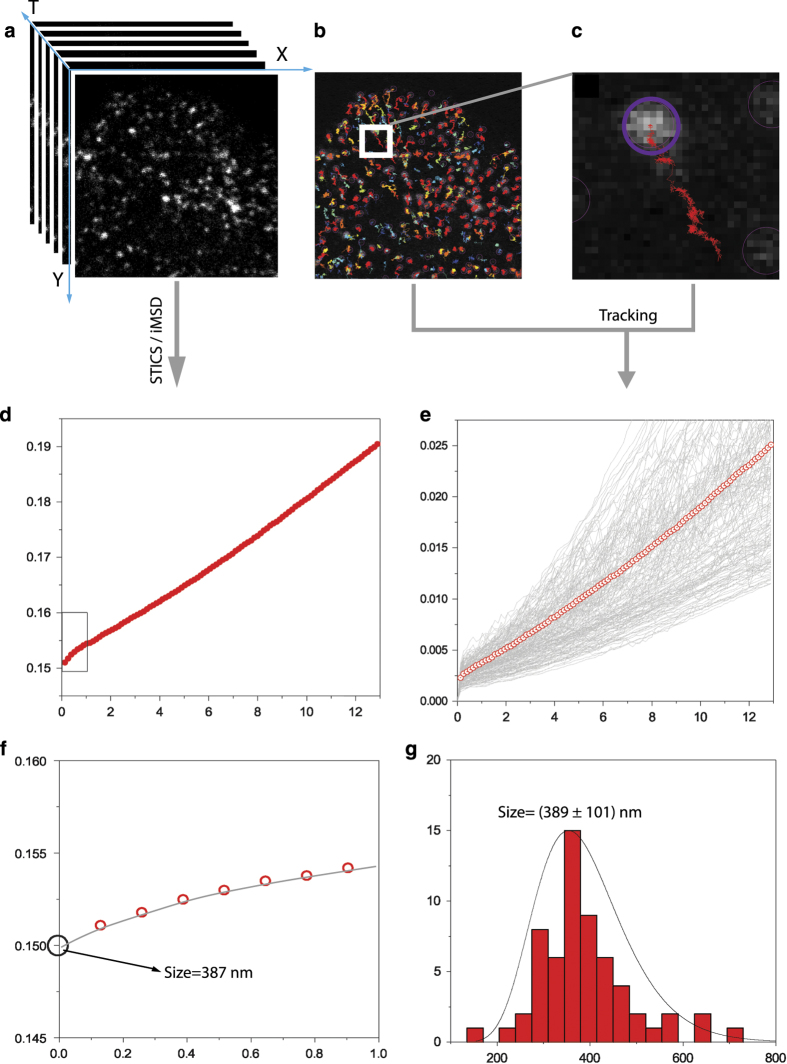
Structural and dynamic parameters derived by SPT and *i*MSD analyses. (**a**) Stack of 1000 intensity images of fluorescently-labelled early endosomes acquired by time-lapse confocal imaging in a living cell at temporal resolution of 129 ms and pixel size of 69 nm. (**b**) Trackmate ImageJ plugin was used to extract trajectories from stack in (**a**). Retrieved trajectories are superimposed on the first frame of the stack. All the trajectories longer than ~12 s were used to calculate the standard MSD with custom MATLAB script. (**c**) Zoomed region from panel (**b**) in which a single trajectory is depicted. (**d**) *i*MSD curve obtained from the provided Matlab algorithm (**e**) All the calculated MSD traces (grey lines) with the average one in red (empty dots). (**f**) Zoomed region of *i*MSD (red empty dots) in (**d**) with relative fitting curve (grey). The square root of the y-axis intercept retrieved by fitting is used to estimate the average size (diameter) of imaged early endosomes. (**g**) Size (diameter) distribution of imaged organelles. Values were extracted from the first frame of the movie (**a**) by means of ImageJ plugin Analyze Particles. This latter allowed to isolate early endosomes fluorescence spots. Then spot diameters were derived from the calculated spot area.

A possible use of the presented datasets is the extraction of structural and/or dynamic parameters by either standard SPT or by the recently proposed *i*MSD algorithm^20^. Comparison between SPT- and *i*MSD-based analyses applied to standard time-lapse imaging is presented in Fig. 3. The former affords, as mentioned above, an impressive amount of detailed information in the form of single-object positions in time and space (i.e. trajectories) captured during the experiment (Fig. 3b and c). The latter, instead, as any other fluctuation-based analysis method, provides average information on *single* diffusing objects, without dwelling on any of them in particular. As such, the *i*MSD plot presented here (Fig. 3d) should be considered as the average diffusion law of all the sub-cellular fluorescent organelles/structures captured by imaging, during the whole acquisition and obtained with no need to extract trajectories. As such, we expect it to be coincident (in terms of the overall shape of the diffusion law) to the standard MSD calculated starting from all single-object trajectories (this is clear from the comparison of traces in Fig. 3d and e). At the high signal-to-noise (S/N) ratios typical of the measurements on fluorescently-labelled organelles/structures (each containing multiple fluorophores), the *i*MSD algorithm would in principle work even on single organelles/structures, thus potentially affording local information on the selected parameters. In addition, as discussed in detail elsewhere^20^, the *i*MSD anlysis affords exclusive access to the estimation of the average organelle size (through the offset or y-axis intercept of the *i*MSD trace) (Fig. 3f). Obtained values are compared to analogous quantification performed by standard image analysis (Fig. 3g).

To help reuse the data by *i*MSD analysis, we made the *i*MSD code available alongside a detailed tutorial on how to use it. The proposed script file was specifically developed to carry out an *i*MSD analysis of fluorescence image time-series. The main goal is to get information about the structure/dynamics of fluorescencently-labeled objects in living cells, through a post-processing of the acquired image-stacks. In the first section of the code, some instrumental and processing parameters must be initialized. Instrumental parameters include: pixel size and frame time (i.e. temporal resolution). Processing parameters define the extent of the spatiotemporal domain for the fitting of the correlation function. Additional variables can be employed to optimize the analysis (e.g. background correction). Technical details about the script execution, the initialization of the input parameters and the expected outputs are given in the tutorial (Data Citation 1). At the end of the computation, results are reported in the command window, depicted in graphical windows and exported in a spreadsheet file. The output (in each of these three forms) contains information about the time evolution of the correlation function, the *i*MSD curve, the corresponding fitting curves (as well as the fitting determination coefficients) and the measured values of the involved dynamic parameters.

**Figure 4:**
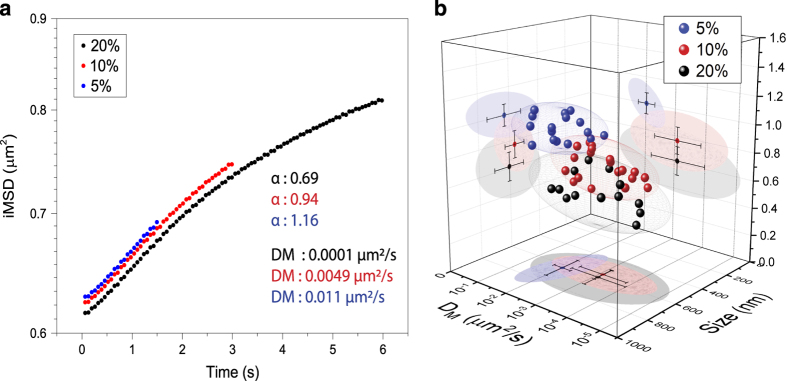
The effect of the experiment time window on *i*MSD analysis. (**a**) Extending the time window of the analysis from 5% (blue curve) to 10% (red, optimal value, see text) and finally 20% (black) of the total duration of the acquired stack of images affects the obtained *i*MSD trace and the corresponding descriptive parameters (mainly α and D_M_ in this example). (**b**) The three sets of data described in (**a**) are here represented in the 3D parametric space (α, size and D_M_) to build the corresponding structural/dynamic fingerprints at the population level.

It should be kept in mind that the outcome of the proposed analysis, analogously to standard SPT, strictly depends on the overall timescale considered. An obvious limit case, on a very large timescale, is the total confinement due to the plasma membrane impenetrable boundary, irrespective of the chosen organelle/structure of interest (not shown). On the other end, on a very short timescale, one may expect to grab the local mode of motion of the structure. To show how this applies to the real case, we present here an application to the lysosome (Fig. 4). Technically, a single time-lapse acquisition (frame rate: 65 ms) was analyzed on three different temporal windows, namely: *i*) a short one (0-1.5 s, 5% of total acquisition), *ii*) an intermediate one (0–3 s, 10% of total acquisition), and *iii*) a long one (0–6 s, 20% of total acquisition) (Fig. 4a). For each of these time windows, the organelle long-range diffusivity (D_M_), the offset parameter (σ_0_^2^, which yield the size, as explained above), and the anomalous-diffusion coefficient (α, which is an indicator of the type of motion of the organelle) can be extracted by fitting the *i*MSD plot (Fig. 4a). As expected, the *i*MSD analysis yields three very different outcomes. In brief, on the shortest timescale, the *i*MSD plot clearly shows an overall super-diffusive trend (i.e. α>1, blue trace in Fig. 4a). Although discernible, the super-diffusion behavior is progressively overwhelmed at the intermediate and then long timescales (α<1, red and black traces in Fig. 4a, respectively), an effect that can be readily ascribed to the influence of the complex intracellular environment on lysosome movement (further biological implications are discussed elsewhere^20^). Overall, the effect is quantitatively depicted at the whole-population level in Fig. 4b, by means of three clusters corresponding to lysosomes acquired at the same frame-rate but extending the time window of the analysis from 5% (blue dots) to 10% (red dots, optimal value, see text) and finally 20% (black dots) of the total acquisition.

In conclusion, from a technological point of view, the *i*MSD algorithm is highly flexible, fully compatible with virtually any kind of imaging modality, from camera-based (e.g. TIRF, SPIM) to scanning-based systems (e.g. confocal, STED-based imaging), from 1- to multi-photon excitation. Also, it can be combined with other analytical tools, either fluctuation-based (e.g. image correlation spectroscopy ICS, spatiotemporal correlation spectroscopy STICS or PLICS^9^) to increase the amount of information that can be extracted from standard imaging, or linked to the use of ‘intelligent’ dyes to probe selected intracellular parameters (e.g. pH, membrane order, etc.).

## Additional information

**How to cite this article**: Ferri, G. *et al*. Time-lapse confocal imaging datasets to assess structural and dynamic properties of subcellular nanostructures. *Sci. Data* 5:180191 doi: 10.1038/sdata.2018.191 (2018).

**Publisher’s note**: Springer Nature remains neutral with regard to jurisdictional claims in published maps and institutional affiliations.

## Supplementary Material



## Figures and Tables

**Table 1:** List of datasets with the corresponding relevant experimental parameters.

**Cell Line**	**Protocol**	**# acquisition**	**# Frame/ acquisition**	**Temporal resolution (ms)**	**Pixel Size (nm)**	**Data set**
HeLa	CellLight Early Endosomes-GFP BacMan 2.0 (Invitrogen)	40	1000	129	69	ee
HeLa	CellLight Late Endosomes-GFP BacMan 2.0 (Invitrogen)	40	1000	129	69	le
HeLa	Lysotracker Red DND-99 (Invitrogen)	26	460	65	69	ly

## References

[d1] Figshare Ferri G. *et al.* 2018 https://doi.org/10.6084/m9.figshare.c.4070033

